# Is there an association between peri-diagnostic vaccination and clinical outcomes in COVID-19 patients?

**DOI:** 10.1017/ash.2023.417

**Published:** 2023-09-08

**Authors:** Julia A. Casazza, Bhaskar Thakur, Trish M. Perl, John J. Hanna, Marlon I. Diaz, Milan Ho, Heather Lanier, Madison Pickering, Sameh N. Saleh, Pankil Shah, Dimpy Shah, Ann Marie Navar, Christoph U. Lehmann, Richard J. Medford, Robert W. Turer

**Affiliations:** 1 UT Southwestern Medical School, Dallas, TX, USA; 2 Clinical Informatics Center, UT Southwestern Medical Center, Dallas, TX, USA; 3 O’Donnell School of Public Health, UT Southwestern Medical Center, Dallas, TX, USA; 4 Department of Internal Medicine, UT Southwestern Medical Center, Dallas, TX, USA; 5 Children’s Hospital of Philadelphia, Philadelphia, PA, USA; 6 Department of Urology, UT Health San Antonio, San Antonio, TX, USA; 7 Department of Population Health Sciences, UT Health San Antonio, San Antonio, TX, USA; 8 Department of Pediatrics, UT Southwestern Medical Center, Dallas, TX, USA; 9 Lyda Hill Department of Bioinformatics, UT Southwestern Medical Center, Dallas, TX, USA; 10 Chief Medical Informatics and Digital Health Officer, ECU Health, Greenville, NC, USA; 11 Department of Emergency Medicine, UT Southwestern Medical Center, Dallas, TX, USA

**Keywords:** COVID-19, vaccination, acute infection, outcomes

## Abstract

**Background:**

Peri-diagnostic vaccination contemporaneous with SARS-CoV-2 infection might boost antiviral immunity and improve patient outcomes. We investigated, among previously unvaccinated patients, whether vaccination (with the Pfizer, Moderna, or J&J vaccines) during the week before or after a positive COVID-19 test was associated with altered 30-day patient outcomes.

**Methods:**

Using a deidentified longitudinal EHR repository, we selected all previously unvaccinated adults who initially tested positive for SARS-CoV-2 between December 11, 2020 (the date of vaccine emergency use approval) and December 19, 2021. We assessed whether vaccination between days –7 and +7 of a positive test affected outcomes. The primary measure was progression to a more severe disease outcome within 30 days of diagnosis using the following hierarchy: hospitalization, intensive care, or death.

**Results:**

Among 60,031 hospitalized patients, 543 (0.91%) were initially vaccinated at the time of diagnosis and 59,488 (99.09%) remained unvaccinated during the period of interest. Among 316,337 nonhospitalized patients, 2,844 (0.90%) were initially vaccinated and 313,493 (99.1%) remained unvaccinated. In both analyses, individuals receiving vaccines were older, more often located in the northeast, more commonly insured by Medicare, and more burdened by comorbidities. Among previously unvaccinated patients, there was no association between receiving an initial vaccine dose between days −7 and +7 of diagnosis and progression to more severe disease within 30 days compared to patients who did not receive vaccines.

**Conclusions:**

Immunization during acute SARS-CoV-2 infection does not appear associated with clinical progression during the acute infectious period.

## Background

Three years after the emergence of SARS-CoV-2, more than 670 million cases of COVID-19 and 6.7 million COVID-19-related deaths have been reported.^1^ Within a year, several SARS-CoV-2 vaccines with different formulations were developed, tested, and found to be efficacious (Pfizer’s BNT162b2, Moderna’s mRNA-1273, and Johnson & Johnson’s Ad26.COV2.S), and made available to adults.

SARS-CoV-2 evades the human immune response by altering protein translation, which reduces the production of interferons and interferon-stimulated gene products.^2,3^ Stimulation of host interferon defenses halts disease progression and shortens hospital stays in infected patients.^4^ Currently, the Centers for Disease Control and Prevention (CDC) and the Advisory Committee on Immunization Practice’s “General Recommendations on Immunization” caution against vaccination during moderate to severe acute illness,^5^ citing that vaccination during this period could cause “diagnostic confusion between manifestations of the underlying illness and effects of vaccination or superimposing adverse effects of the vaccine on the underlying illness.”^6^ However, data supporting the rationale that acute illness may alter outcomes are limited. In the setting of most vaccine-preventable infections (including influenza), vaccines rarely cause high fever.^7,8^ The COVID-19 vaccine is a notable exception, as these vaccines targeted toward SARS-CoV-2 stimulate side effects similar to an acute phase reactant.^9,10^ While fever from a vaccine might affect whether to return to work or other clinical decision-making, there have not been studies formally evaluating the safety of vaccination in the period immediately surrounding COVID-19 illness.

Therapeutic vaccination is a strategy that relies upon the immune response induced by immunization to alter the disease course in a sick patient. Therapeutic vaccination is an active area of investigation in Hepatitis B^6^ and HIV^7^ research, with therapeutic vaccines shown to be safe in chronically infected individuals.^8,9^ Case reports documenting curative therapeutic vaccination for arteritis caused by *P. insidiosum* exist,^10^ and postexposure prophylaxis with inactivated rabies vaccine has eliminated cases in the US among those treated.^11,12^ However, CDC guidelines advocate against vaccination during COVID-19 illness despite the absence of prior studies assessing the effect of vaccination in the immediate peri-diagnostic period on clinical outcomes.

Earlier treatment with exogenous interferon-beta, which accelerates immune response, correlates with shorter duration of illness and lower ICU admission rates in hospitalized COVID-19 patients.^13,14^ Accordingly, we raised the question of whether vaccination during acute COVID-19 infection might improve clinical outcomes by stimulating alternative pathways for the immune system to fight infection as demonstrated previously for other diseases that evade immunity.^15^

Exploring the association between peri-diagnostic vaccination and clinical outcomes is challenging because clinicians rarely ignore CDC guidelines recommending against the practice. Accordingly, single-site studies would not provide an adequate sample size to explore this question. A large, national database, however, might provide sufficient samples of this rare event to facilitate exploration. This retrospective observational study harnessing a large, national database of patients diagnosed with COVID-19 across the United States serves as a first line of inquiry into this important research question.

## Methods

### Objective

This study evaluated the effect of COVID-19 vaccination immediately prior to or following diagnosis of COVID-19 (either by PCR or positive antigen test).

### Study design

We conducted an observational retrospective cohort study using a national repository of electronic health record (EHR) data to explore the relationship between peri-diagnostic vaccination and clinical outcomes in adult patients with COVID-19 infection. The overall population of interest consisted of previously unvaccinated patients over 18 who were diagnosed with COVID-19 via polymerase chain reaction (PCR) or antigen testing between December 11, 2020 (the date of vaccine emergency use approval in the United States) and December 19, 2021 (the last day for which we had 30-day follow-up data). Patients without documented vaccination up to seven days prior to infection were considered unvaccinated. To minimize the effects of immune memory from prior infection, we only included the first documented COVID-19 infection within the database for each patient included in our study.

From this population, the exposure group consisted of patients who received their first COVID-19 vaccination within 7 days before or after diagnosis. We examined vaccinations given during this period based on data that suggest the pooled incubation period for all COVID-19 variants is approximately 7 days.^16^ As date of symptom onset was not logged in the Optum database, choosing a cutoff of 7 days on either side of a positive test allows us to examine patients who were vaccinated earlier in their illness course while also confining vaccination to the period of acute infection. The control group consisted of patients who remained unvaccinated for 30 days following diagnosis. Included vaccines were Pfizer’s BnT162b2, Moderna’s mRNA-1273, and Johnson & Johnson’s Ad26.COV.S. The database reported vaccinations administered by contributing sites as well as patient-reported vaccinations; we included both in this study. Initial COVID-19 diagnosis for both groups was defined by the first instance of a positive PCR or antigen test. Patients were ineligible for the study if they had received at least one dose of any of the candidate vaccines more than 7 days prior to their initial positive COVID-19 test.

The primary outcome was clinical progression of COVID-19 disease during days 8–30 following diagnosis. We conducted two analyses: one for patients not hospitalized at diagnosis and another for patients hospitalized at diagnosis due to differing definitions of clinical progression. For nonhospitalized patients, progression was defined as admission to the inpatient or intensive care units. For hospitalized patients, progression was defined as admission to the intensive care unit. We excluded patients who were admitted to intensive care at the time of diagnosis. We did not include outcomes on days 0–7 because this would overlap with the period during which vaccination was permitted, meaning that some outcomes would be logged before our exposure (vaccination) occurred. Further, all human studies exploring COVID-19 vaccine effectiveness started on day 7 or 14 following the first dose of vaccine.^17^ Among nonhospitalized patients, outcomes observed were transition to hospitalization and/or intensive care. We identified hospitalizations based on patient classification as “observation,” “inpatient,” or, alternatively, “other patient type” with a procedure or medication specified by the WHO-Clinical Progression Scale.^18^ Patients requiring endotracheal intubation, mechanical ventilation, tracheostomy placement, high flow nasal cannula, and noninvasive positive pressure ventilation without a documented intensive care unit (ICU) stay were included in the intensive care severity category. Because all data were deidentified, no institutional review board approval was required for this study.

### Data source

We used Optum’s longitudinal EHR repository, which is derived from dozens of healthcare organizations in the United States and includes patients’ healthcare data from more than 700 hospitals and 7,000 clinics (Optum Inc., Eden Prairie, MN, USA). The data are certified as deidentified by an independent statistical expert following HIPAA statistical deidentification rules and managed according to Optum’s customer data use agreements. The COVID-19 data set incorporates a wide swath of raw clinical data, including new, unmapped COVID-specific clinical data from inpatient and ambulatory EHRs, practice management systems, and numerous other internal systems. Information is processed from across the continuum of care, including acute inpatient stays and outpatient visits. The COVID-19 data captures point-of-care diagnostics specific to the COVID-19 patient during initial presentation, acute illness, and convalescence with over 500 mapped labs and bedside observations, including COVID-19-specific testing. The Optum COVID-19 data elements include patient-level information like demographics, hospitalizations, Emergency Department (ED) visits, intensive care unit (ICU) admissions, mortality, medications, laboratory tests, procedures, and diagnoses. The data are comprised of multiple tables linked by a common patient identifier (an anonymous, randomized string of characters).

### Statistical analysis

For patients hospitalized (but not receiving intensive care) between days 0 and 7 after diagnosis, we used binary logistic regression to evaluate progression to ICU-level care on days 8–30. For patients not hospitalized during days 0–7, we used proportional odds logistic regression to evaluate progression as an ordinal outcome including hospitalization and ICU level care on days 8–30. Ordinality was assessed in advance using the Wald test for parallel lines. Due to how deaths are registered in the Optum database (all deaths, regardless of actual date, are recorded as having occurred on the first of the month), deaths were not included in the analysis. Assuming a significant effect in the unadjusted models, we performed propensity score matching using the following covariates: age, sex, race, ethnicity, region, body mass index (BMI), insurance status, and past medical history using individual elements from the Charlson Comorbidity Index. Overall model performance was evaluated compared to the null model using likelihood ratio χ^2^ tests. Alpha was set to 0.05 for significance testing. Statistical analysis was performed using Stata version 17, Parallel Edition (StataCorp, College Station, TX, USA).

## Results

**Figure 1.**
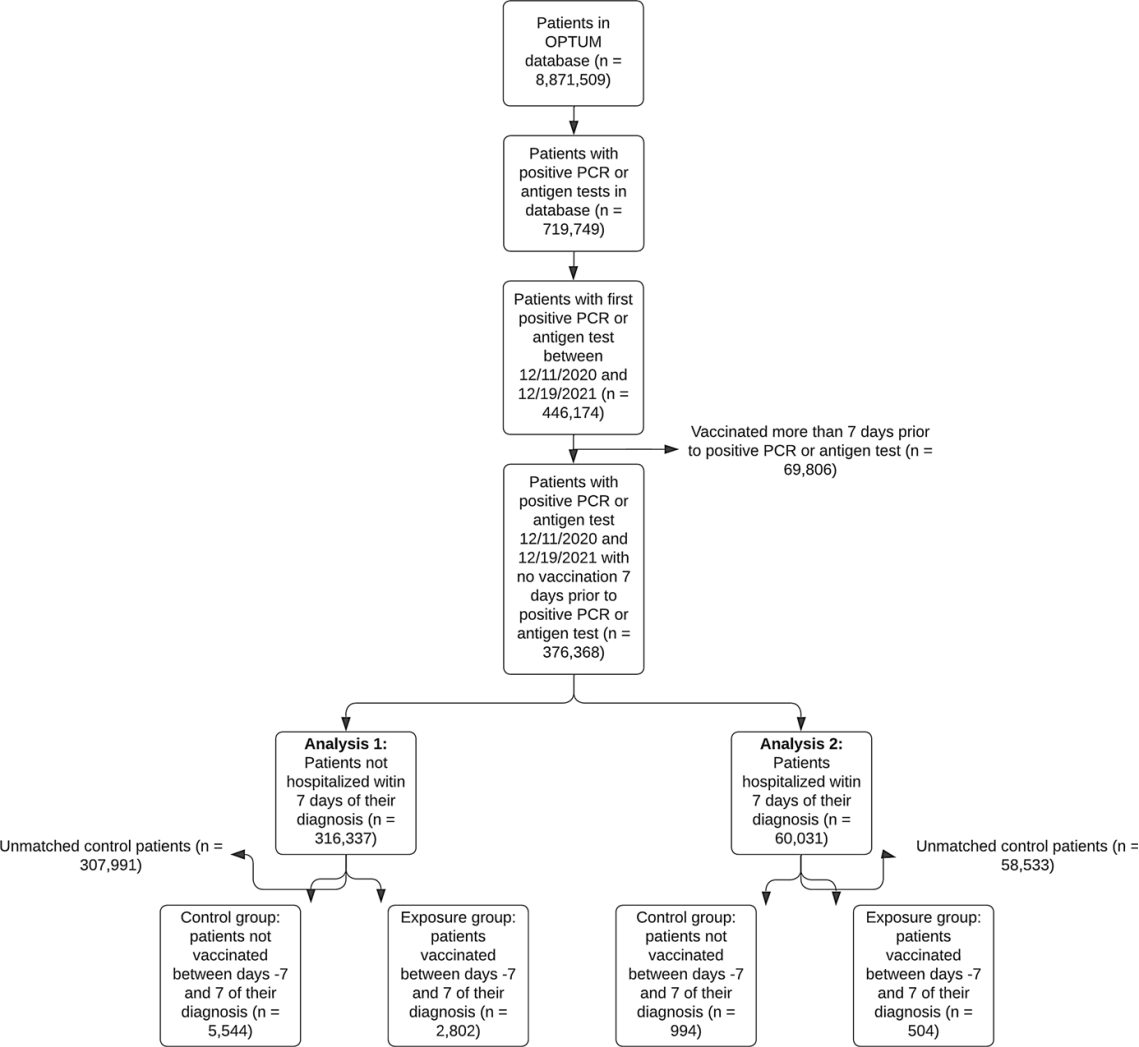
CONSORT diagram illustrating how patients were selected from the OPTUM database for inclusion in the study.

The Optum database contains information on 8,871,509 patients. These patients had a mean age of 47 [St. Dev. 22.9] years. Patients were largely female (56.1%), White (67.1%), and non-Hispanic (77.1%). Patients lived in the Midwest (41.6%), Northeast (21.6%), South (14.9%), and West (9.3%) (Supplementary Table S1). As of January 19, 2022 (the last date for which data were available), 2,056,878 patients had at least one dose of the three approved vaccines documented. A CONSORT diagram of patient selection during the study is depicted in Fig. 1.

Among hospitalized patients, those who received peri-diagnostic vaccinations were older (mean age 61.6 [17.6] vs 56.6 [19.2], *P* < .001), less often Hispanic (12.9% vs 16.9%, *P* = .044), more commonly located in the Northeast (23.4% vs 14.1%, *P* < .001), and more commonly insured by Medicare (32.8% vs 25.5%, *P* < .001) or commercial payors (43.5% vs 41.8%, *P* < .001) (Table 1). Among nonhospitalized patients, those receiving peri-diagnostic vaccinations were older (mean age 49.2 [17.0] vs 45.4 [17.2], *P* < .001), more often female (63.7% vs 55.8%, *P* < .001), White (71.4% vs 64.1%, *P* < .001), and more commonly insured by Medicare (12.5% vs 8.9%, *P* < .001) or commercial payors (64.4% vs 52.9%, *P* < .001) (Table 2). The prevalence of illnesses measured in the Charlson Comorbidity Index was higher among vaccinated patients in both groups (Tables 1 and 2).

**Table 1.** Characteristics of all patients hospitalized between day 0 and day 7 postinfection

		**Not vaccinated (** ***n*** **=59,488)**	**Vaccinated (** ***n*** **=543)**	*P* **-value**
Demographic variables				
Patient age, mean (SD)		56.6 (19.2)	61.6 (17.6)	<.001
Gender	Male	27,152 (45.6%)	255 (47.0%)	.14
	Female	32,316 (54.3%)	287 (52.9%)	
	Unknown	20 (<1%)	1 (0.2%)	
Race	African American	8,842 (14.9%)	85 (15.7%)	.86
	Asian	1,359 (2.3%)	10 (1.8%)	
	Caucasian	40,418 (67.9%)	369 (68.0%)	
Ethnicity	Other/Unknown	8,869 (14.9%)	79 (14.5%)	
	Hispanic	10,034 (16.9%)	70 (12.9%)	.044
	Not Hispanic	43,722 (73.5%)	421 (77.5%)	
	Unknown	5732 (9.6%)	52 (9.6%)	
Region	Midwest	15,172 (25.5%)	183 (33.7%)	<.001
	Northeast	10,199 (17.1%)	127 (23.4%)	
	Other/Unknown	5,324 (8.9%)	45 (8.3%)	
	South	27,170 (45.7%)	170 (31.3%)	
	West	1,623 (2.7%)	18 (3.3%)	
BMI, mean (SD)		30.9 (7.0)	31.3 (6.9)	.31
Insurance	Commercial	24,867 (41.8%)	236 (43.5%)	<.001
	Medicaid	7,069 (11.9%)	53 (9.8%)	
	Medicare	15,152 (25.5%)	178 (32.8%)	
	Other payor type	3,251 (5.5%)	23 (4.2%)	
	Uninsured	3,536 (5.9%)	15 (2.8%)	
	Unknown	5,613 (9.4%)	38 (7.0%)	
Comorbidities				
Myocardial infarction	Absent	50,617 (85.1%)	437 (80.5%)	.003
	Present	8,871 (14.9%)	106 (19.5%)	
Congestive heart failure	Absent	47,878 (80.5%)	373 (68.7%)	<.001
	Present	11,610 (19.5%)	170 (31.3%)	
Peripheral vascular disease	Absent	51,339 (86.3%)	415 (76.4%)	<.001
	Present	8,149 (13.7%)	128 (23.6%)	
Cerebrovascular disease	Absent	49,283 (82.8%)	406 (74.8%)	<.001
	Present	10,205 (17.2%)	137 (25.2%)	
Dementia	Absent	54,965 (92.4%)	487 (89.7%)	.018
	Present	4,523 (7.6%)	56 (10.3%)	
COPD	Absent	41,939 (70.5%)	327 (60.2%)	<.001
	Present	17,549 (29.5%)	216 (39.8%)	
Rheumatic diseases	Absent	56,930 (95.7%)	529 (97.4%)	.049
	Present	2,558 (4.3%)	14 (2.6%)	
Peptic ulcer disease	Absent	56,893 (95.6%)	511 (94.1%)	.083
	Present	2,595 (4.4%)	32 (5.9%)	
Mild liver disease	Absent	52,650 (88.5%)	470 (86.6%)	.16
	Present	6,838 (11.5%)	73 (13.4%)	
Diabetes mellitus, uncomplicated	Absent	40,500 (68.1%)	337 (62.1%)	.003
	Present	18,988 (31.9%)	206 (37.9%)	
Diabetes mellitus, complicated	Absent	48,830 (82.1%)	512 (94.3%)	<.001
	Present	10,658 (17.9%)	31 (5.7%)	
Kidney disease	Absent	47,439 (79.7%)	382 (70.3%)	<.001
	Present	12,049 (20.3%)	161 (29.7%)	
Malignancy	Absent	52,887 (88.9%)	456 (84.0%)	<.001
	Present	6,601 (11.1%)	87 (16.0%)	
Severe liver disease	Absent	58,587 (98.5%)	536 (98.7%)	.67
	Present	901 (1.5%)	7 (1.3%)	
AIDS	Absent	59,084 (99.3%)	538 (99.1%)	.50
	Present	404 (0.7%)	5 (0.9%)	
Metastatic solid tumor	Absent	57,620 (96.9%)	518 (95.4%)	.052
	Present	1,868 (3.1%)	25 (4.6%)	

**Table 2.** Characteristics of all patients not hospitalized between days 0 to 7 postinfection

		**Not vaccinated(** ***n*** **=313,493)**	**Vaccinated (** ***n*** **=2,844)**	*P* **-value**
Demographic variables				
Patient age, mean (SD)		45.4 (17.2)	49.2 (17.0)	<.001
Gender	Male	138,382 (44.1%)	1,028 (36.1%)	<.001
	Female	174,802 (55.8%)	1,813 (63.7%)	
	Unknown	309 (0.1%)	3 (0.1%)	
Race	African American	31,309 (10.0%)	201 (7.1%)	<.001
	Asian	4,397 (1.4%)	64 (2.3%)	
	Caucasian	200,868 (64.1%)	2,032 (71.4%)	
	Other/Unknown	76,919 (24.5%)	547 (19.2%)	
Ethnicity	Hispanic	31,422 (10.0%)	271 (9.5%)	.087
	Not Hispanic	243,843 (77.8%)	2,189 (77.0%)	
	Unknown	38,228 (12.2%)	384 (13.5%)	
Region	Midwest	118,109 (37.7%)	1,044 (36.7%)	<.001
	Northeast	52,542 (16.8%)	761 (26.8%)	
	Other/Unknown	54,001 (17.2%)	347 (12.2%)	
	South	69,111 (22.0%)	535 (18.8%)	
	West	19,730 (6.3%)	157 (5.5%)	
BMI, mean (SD)		30.5 (5.9)	30.7 (6.1)	.077
Insurance	Commercial	165,691 (52.9%)	1,831 (64.4%)	<.001
	Medicaid	29,658 (9.5%)	148 (5.2%)	
	Medicare	27,884 (8.9%)	355 (12.5%)	
	Other payor type	16,871 (5.4%)	87 (3.1%)	
	Uninsured	13,873 (4.4%)	57 (2.0%)	
	Unknown	59,516 (19.0%)	366 (12.9%)	
Comorbidities				
Myocardial infarction	Absent	303,843 (96.9%)	2,699 (94.9%)	<.001
	Present	9,650 (3.1%)	145 (5.1%)	
Congestive heart failure	Absent	300,591 (95.9%)	2,670 (93.9%)	<.001
	Present	12,902 (4.1%)	174 (6.1%)	
Peripheral vascular disease	Absent	300,561 (95.9%)	2,679 (94.2%)	<.001
	Present	12,932 (4.1%)	165 (5.8%)	
Cerebrovascular disease	Absent	299,058 (95.4%)	2,645 (93.0%)	<.001
	Present	14,435 (4.6%)	199 (7.0%)	
Dementia	Absent	310,374 (99.0%)	2,806 (98.7%)	.068
	Present	3,119 (1.0%)	38 (1.3%)	
COPD	Absent	253,023 (80.7%)	2,200 (77.4%)	<.001
	Present	60,470 (19.3%)	644 (22.6%)	
Rheumatic diseases	Absent	306,542 (97.8%)	2,824 (99.3%)	<.001
	Present	6,951 (2.2%)	20 (0.7%)	
Peptic ulcer disease	Absent	308,457 (98.4%)	2,778 (97.7%)	.003
	Present	5,036 (1.6%)	66 (2.3%)	
Mild liver disease	Absent	296,131 (94.5%)	2,634 (92.6%)	<.001
	Present	17,362 (5.5%)	210 (7.4%)	
Diabetes mellitus, uncomplicated	Absent	275,215 (87.8%)	2,390 (84.0%)	<.001
	Present	38,278 (12.2%)	454 (16.0%)	
Diabetes mellitus, complicated	Absent	298,624 (95.3%)	2,794 (98.2%)	<.001
	Present	14,869 (4.7%)	50 (1.8%)	
Kidney disease	Absent	297,811 (95.0%)	2,642 (92.9%)	<.001
	Present	15,682 (5.0%)	202 (7.1%)	
Malignancy	Absent	297,932 (95.0%)	2,600 (91.4%)	<.001
	Present	15,561 (5.0%)	244 (8.6%)	
Severe liver disease	Absent	312,534 (99.7%)	2,834 (99.6%)	.66
	Present	959 (0.3%)	10 (0.4%)	
AIDS	Absent	312,645 (99.7%)	2,838 (99.8%)	.54
	Present	848 (0.3%)	6 (0.2%)	
Metastatic solid tumor	Absent	310,170 (98.9%)	2,803 (98.6%)	.048
	Present	3323 (1.1%)	41 (1.4%)	

Among hospitalized patients, there were 543 patients (0.9%) who were vaccinated between days −7 and 7 and 59,488 (99.1%) patients who were not vaccinated. Progression was observed in seven vaccinated patients (1.29%) and 497 unvaccinated patients (0.84%). The binary logistic regression (LR) model evaluating clinical progression as a function of vaccination had a nonsignificant LR χ^2^ test (*P* = .232). Among nonhospitalized patients, there were 2,844 (0.9%) of patients vaccinated between days −7 and 7, and 313,493 (99.1%) were not vaccinated. The data set had a Wald test of parallel lines assumption that was not significant (*P* = .987), which suggests that the proportional odds/parallel lines assumption was not violated, supporting the use of a proportional odds model (Table 3). The proportional odds logistic regression model evaluating clinical progression as a function of vaccination among nonhospitalized patients also had a nonsignificant LR χ^2^ test (*P* = .643) (Table 3).

**Table 3.** Rates of progression in hospitalized and nonhospitalized patients

Analysis	Measures	Hospitalized		Nonhospitalized	
		Vaccinated, progression	Not vaccinated, progression	Vaccinated, progression	Not vaccinated, progression
Unmatched cohort	Number (%) of patients	7/543 (1.29%)	497/59,488 (0.84%)	46/2,844 (1.62%)	4,747/313,493 (1.51%)
	OR (95% CI)	1.00 (reference)	0.64 (0.30, 1.37)	1.00 (reference)	0.93 (0.70, 1.25)
	*P*-value	0.23, 0.25^*^		0.643, 0.65^*^	
Matched cohort	Number (%) of patients	7/504 (1.39)	3/994 (0.30)	46/2,802 (1.64%)	103/5,544 (1.86%)
	OR (95% CI)	1.00 (reference)	0.21 (0.05, 0.83)	1.00 (reference)	1.13 (0.80, 1.61)
	*P*-value	0.037, 0.026^*^		0.54, 0.48^*^	

* Indicates *P*-value reported for OR.

With no significant difference observed in unadjusted models, we then performed propensity score-matched analysis to explore whether cohorts comparable in demographic and comorbid factors experienced any clinical difference following vaccination. In hospitalized and nonhospitalized patients, propensity score matching yielded cohorts that were statistically indistinguishable regarding demographic data and rates of comorbidities (Supplementary Tables S2 and S3). Among hospitalized patients, clinical progression was more common in those who were vaccinated than those who were not (Table 3; 1.39% vs 0.30%). Relative to vaccinated patients, unvaccinated patients had an OR of progression of 0.21 (95% CI 0.05–0.83) (Table 3). Among nonhospitalized patients, clinical progression did not differ between those who were and were not vaccinated peri-diagnostically (Table 3).

## Discussion

In this observational study, we evaluated whether peri-diagnostic vaccination was associated with worse clinical outcomes among adult patients with COVID-19. Patients who received peri-diagnostic vaccinations were more often White, older, more commonly located in the Northeast, more commonly insured by Medicare or commercial firms, and more burdened by comorbidities. As Optum only logs month, not date of death, this outcome was not assessed in our analysis. Our partial proportional odds model showed that vaccination between days −7 and 7 of a positive test was not associated with clinical progression in patients, regardless of whether they were hospitalized at the outset. However, analysis of propensity-score matched vaccinated and not vaccinated groups showed that vaccination was associated with clinical progression to a higher level of care during days 8–30 postinfection in hospitalized patients. This result contradicts our hypothesis that peri-diagnostic vaccination would decrease rates of clinical progression. It is important to note the number of patients included in our PSM analysis who progressed from hospitalization to ICU care was very limited in vaccinated and not vaccinated groups (7 and 3, respectively), making interpretation of this result difficult. Vaccination was not associated with clinical progression to a higher level of care in ambulatory patients.

Multiple theories could explain the limited association between peri-diagnostic vaccinations on clinical progression. Acute-phase SARS-CoV-2 infection causes lymphopenia,^19^ which could impair the immune response to vaccination. SARS-CoV-2 vaccines largely work by stimulating plasma cell production of broadly neutralizing antibodies, which are not produced in large quantities before most acute infections resolve.^20^ Though exposure to spike protein from vaccination induces innate immune responses (as evidenced by vaccine-induced fever), our results suggest that these responses are not sufficient in the context of acute illness to meaningfully alter outcomes. Study in humans shows that, though levels of bloodstream inflammatory monocytes and expression of genes encoding interferons increase 1–2 days following an initial immunization against SARS-CoV-2, the induction of innate immune responses with the second vaccine dose is several-fold greater.^21^

As COVID-19 moves from pandemic to endemic, host-directed therapies are needed to protect from severe illness caused by new viral variants. Our results suggest that the innate immune stimulation from first-dose vaccination is likely not sufficient to meaningfully alter clinical outcomes. Approached differently, the fact that vaccines do not meaningfully alter clinical course can be leveraged to promote vaccine uptake among unvaccinated (or undervaccinated) patients when hospitalized with COVID-19 illness. Nearly 3 years into the pandemic, 22% of American adults remain unwilling to obtain COVID-19 vaccines.^22^ Nonetheless, more research is needed to elucidate specific mechanisms of innate immunity that allow patients to promptly recover from COVID-19. Peri-diagnostic vaccination, aided by adjuvants, could have a role in fighting SARS-CoV-2 and other viral illnesses in the future, but our results suggest that, given the natural history of COVID-19 and the time taken for vaccines to stimulate adaptive immunity, currently available vaccines are unlikely to meaningfully affect outcomes when administered immediately after diagnosis.

### Limitations

Our study, like any study using a large national EHR data repository, suffers from limitations. Evaluated 2 weeks following the initial dose, the three vaccines employed in our study (mRNA-1273, BNT162b2, and Ad26.COV2.S) induce CD8 T cell responses at similar rates, but mRNA-1273 and BNT162b2 stimulate higher titers of neutralizing antibodies than Ad26.COV2.S.^23^ Further study stratifying results based on vaccine type might identify differences in efficacy between vaccines in the peri-diagnostic period; unfortunately, our data collection did not allow us to distinguish between vaccine types. Among vaccinated individuals, the difference in the interval between diagnosis and vaccination might affect outcomes, and future analyses should further interrogate this question. Interpretation of our results is limited by the diversity of individuals in our cohort, particularly the low representation of Hispanic/Latino patients (Table S1). The median ages of patients included in our analysis of nonhospitalized and hospitalized patients were approximately 49 and 61, limiting the applicability of our results to older patients, who are the most vulnerable to SARS-CoV-2 infection.^24^ Differences in care, such as variability among hospitals in the use of steroids and remdesivir during early infection, likely affected clinical outcomes in our hospitalized group. Since peri-diagnostic vaccination is against current guidelines, the exposure of interest was very rare among the population included in the database. This very small sample size is a significant limitation and a larger sample might show a different signal. Unfortunately, such a sample is unlikely to be identified given the rarity of peri-diagnostic vaccination. Viewed another way, the size of the Optum database is a strength since it allowed us to perform this study in the first place. We had a low rate of missing comorbidities, however, this number may have been falsely low, especially for patients with limited entries in the database. Despite the limitations that come with studying a rare, complex event in the context of a public health emergency, our study provides further evidence for the CDC’s recommendation against vaccination shortly following infection and generates intriguing questions regarding the ideal vaccination timeline.

## Conclusions

In this retrospective observational cohort study, we did not observe an association between vaccination for COVID-19 during acute illness and severity outcomes in unmatched cohort analysis. When cohorts were matched for demographic and comorbid conditions, vaccination was associated with greater odds of clinical progression. Despite the large size of the Optum electronic health record data set, vaccination was rare in the acute phase, and our sample size was limited. Further studies using other national and international data sets are recommended to further evaluate this important clinical question.

## Supporting information

Casazza et al. supplementary material Casazza et al. supplementary material

## Supplementary material

For supplementary material accompanying this paper visit http://doi.org/10.1017/ash.2023.417. click here to view supplementary material

## References

[1] COVID-19 Map – Johns Hopkins Coronavirus Resource Center [Internet]. [cited 2021 Sep 12]. https://coronavirus.jhu.edu/map.html

[2] Siu K-L , Chan C-P , Kok K-H , Woo PC-Y , Jin D-Y. Suppression of innate antiviral response by severe acute respiratory syndrome coronavirus M protein is mediated through the first transmembrane domain. Cell Mol Immunol [Internet] 2014 [cited 2021 Sep 12];11:141. pmc/articles/PMC4003381/ 24509444 10.1038/cmi.2013.61 PMC4003381

[3] Hsu JC-C , Laurent-Rolle M , Pawlak JB , Wilen CB , Cresswell P. Translational shutdown and evasion of the innate immune response by SARS-CoV-2 NSP14 protein. Proc Natl Acad Sci [Internet] 2021 [cited 2021 Sep 12];118. https://www.pnas.org/content/118/24/e2101161118 10.1073/pnas.2101161118 PMC8214666 34045361

[4] Nakhlband A , Fakhari A , Azizi H. Interferon-beta offers promising avenues to COVID-19 treatment: a systematic review and meta-analysis of clinical trial studies. Naunyn Schmiedebergs Arch Pharmacol [Internet] 2021 [cited 2021 Sep 12];394:1. /pmc/articles/PMC7883756/ 33587164 10.1007/s00210-021-02061-x PMC7883756

[5] Services H. General recommendations on immunization. J Am Med Assoc 2011;262:339–340.

[6] Brady MT , Byington CL , Davies HD , et al. HPV vaccine recommendations. Pediatrics [Internet] 2012 [cited 2022 Jul 15];129:602–605. /pediatrics/article/129/3/602/31694/HPV-Vaccine-Recommendations 22371460 10.1542/peds.2011-3865

[7] 372 Fluzone® High-Dose LE7228 HIGHLIGHTS OF PRESCRIBING INFORMATION. 2018 [cited 2022 Oct 25]. www.vaers.hhs.gov

[8] Highlights of Prescribing Information. [cited 2022 Oct 25]. www.vaers.hhs.gov

[9] Fda, Cber. Package Insert – COMIRNATY (purple cap). [cited 2022 Oct 25]. http://vaers.hhs.gov

[10] FACT SHEET FOR HEALTHCARE PROVIDERS ADMINISTERING VACCINE (VACCINATION PROVIDERS). [cited 2022 Oct 25]. www.modernatx.com/covid19vaccine-

[11] Ugolini G , Hemachudha T. Rabies: changing prophylaxis and new insights in pathophysiology. Curr Opin Infect Dis [Internet] 2018 [cited 2022 Oct 25];31:93–101. https://journals.lww.com/co-infectiousdiseases/Fulltext/2018/02000/Rabies__changing_prophylaxis_and_new_insights_in.14.aspx 29293476 10.1097/QCO.0000000000000420

[12] Ahmad O , Moor-Smith M , Hasselback P. Just the facts: rabies post-exposure prophylaxis. Can J Emerg Med [Internet] 2021 [cited 2022 Oct 25];23:153–155. https://link.springer.com/article/10.1007/s43678-020-00018-2 10.1007/s43678-020-00018-2 33709368

[13] Rahmani H , Davoudi-Monfared E , Nourian A , et al. Interferon β-1b in treatment of severe COVID-19: A randomized clinical trial. Int Immunopharmacol [Internet] 2020 [cited 2023 Jan 16];88. https://pubmed.ncbi.nlm.nih.gov/32862111/ 10.1016/j.intimp.2020.106903 PMC7445008 32862111

[14] Alavi Darazam I , Shokouhi S , Pourhoseingholi MA , et al. Role of interferon therapy in severe COVID-19: the COVIFERON randomized controlled trial. Sci Rep [Internet] 2021 [cited 2023 Jan 16];11. https://pubmed.ncbi.nlm.nih.gov/33850184/ 10.1038/s41598-021-86859-y PMC8044200 33850184

[15] Thitithanyanont A , Mendoza L , Chuansumrit A , et al. Use of an immunotherapeutic vaccine to treat a life-threatening human arteritic infection caused by Pythium insidiosum. Clin Infect Dis [Internet] 1998 [cited 2022 May 30];27:1394–1400. https://pubmed.ncbi.nlm.nih.gov/9868649/ 9868649 10.1086/515043

[16] Wu Y , Kang L , Guo Z , Liu J , Liu M , Liang W. Incubation period of COVID-19 caused by unique SARS-CoV-2 strains: a systematic review and meta-analysis. JAMA Netw Open [Internet] 2022 [cited 2023 Feb 7];5:e2228008–e2228008. https://jamanetwork.com/journals/jamanetworkopen/fullarticle/2795489 35994285 10.1001/jamanetworkopen.2022.28008 PMC9396366

[17] Sadarangani M , Marchant A , Kollmann TR. Immunological mechanisms of vaccine-induced protection against COVID-19 in humans. Nat Rev Immunol [Internet] 2021;21:475–484. 10.1038/s41577-021-00578-z 34211186 PMC8246128

[18] Marshall JC , Murthy S , Diaz J , et al. Personal view a minimal common outcome measure set for COVID-19 clinical research. Lancet Infect Dis [Internet] 2020 [cited 2022 Jun 9]. www.thelancet.com/infectionPublishedonline 10.1016/S1473-3099(20)30483-7 PMC7292605 32539990

[19] Chen G , Wu D , Guo W , et al. Clinical and immunological features of severe and moderate coronavirus disease 2019. J Clin Invest 2020;130:2620–2629. 32217835 10.1172/JCI137244 PMC7190990

[20] Mascellino MT , Oliva A. Overview of the main anti-SARS-CoV-2 vaccines: mechanism of action, efficacy and safety. Infect Drug Resist 2021;14:3459–3476. 34511939 10.2147/IDR.S315727 PMC8418359

[21] Arunachalam PS , Scott MKD , Hagan T , et al. Systems vaccinology of the BNT162b2 mRNA vaccine in humans. Nat 2021 5967872 [Internet] 2021 [cited 2023 Feb 7];596:410–416. https://www.nature.com/articles/s41586-021-03791-x 10.1038/s41586-021-03791-x PMC8761119 34252919

[22] The Morning Consult COVID-19 Vaccine Dashboard [Internet]. [cited 2023 Feb 26]. https://morningconsult.com/covid19-vaccine-dashboard/

[23] Zhang Z , Mateus J , Coelho CH , et al. Humoral and cellular immune memory to four COVID-19 vaccines. Cell [Internet] 2022 [cited 2023 Jan 16];185:2434–2451.e17. http://www.cell.com/article/S0092867422006535/fulltext 35764089 10.1016/j.cell.2022.05.022 PMC9135677

[24] Crimmins EM. Age-related vulnerability to Coronavirus disease 2019 (COVID-19): biological, contextual, and policy-related factors. Public Policy Aging Rep [Internet] 2020 [cited 2022 May 23];30:142–146. https://academic.oup.com/ppar/article/30/4/142/5902126 33214754 10.1093/ppar/praa023 PMC7499698

