# Supplementary material for: Is there an association between peri-diagnostic vaccination and clinical outcomes in COVID-19 patients?
Source: Antimicrob Steward Healthc Epidemiol. 2023 Sep 8;3(1):e150. doi: 10.1017/ash.2023.417 (PMC10523550; doi:10.1017/ash.2023.417)
Supplement: Casazza et al. supplementary material [file S2732494X23004175sup001.docx]

## Supplementary Tables

Supplementary Table S1: All Patient Characteristics in Optum database

| Factor |  |  |
| --- | --- | --- |
| Patient Age, mean (SD) |  | 47.0 (22.9) |
| Patient BMI, mean (SD) |  | 28.8 (8.4) |
| Gender | Male | 3,872,259 (43.6%) |
|  | Female | 4,978,075 (56.1%) |
|  | Unknown | 21,175 (0.2%) |
| Race | African American | 883,666 (10.0%) |
|  | Asian | 178,655 (2.0%) |
|  | Caucasian | 5,951,268 (67.1%) |
|  | Other/Unknown | 1,857,920 (20.9%) |
| Ethnicity | Hispanic | 613,547 (6.9%) |
|  | Not Hispanic | 6,837,447 (77.1%) |
|  | Unknown | 1,420,515 (16.0%) |
| Region | Midwest | 3,695,215 (41.7%) |
|  | Northeast | 1,913,703 (21.6%) |
|  | Other/Unknown | 1,109,057 (12.5%) |
|  | South | 1,325,362 (14.9%) |
|  | West | 828,172 (9.3%) |
| Insurance (most recent entry) | Commercial | 4,110,128 (54.9%) |
|  | Medicaid | 951,837 (12.7%) |
|  | Medicare | 1,319,466 (17.6%) |
|  | Other Payor Type | 418,179 (5.6%) |
|  | Uninsured | 273,416 (3.7%) |
|  | Unknown | 405,608 (5.4%) |

Supplementary Table S2: Characteristics of all patients hospitalized between day 0 and day 7 post-infection included in propensity score matched analysis

|  |  | Not vaccinated  (n = 994) | Vaccinated (n = 504) | p-value |
| --- | --- | --- | --- | --- |
| Demographic variables | | | | |
| Patient age, mean (SD) |  | 61.5 (18.6) | 62.1 (17.5) | 0.59 |
| Gender | Male | 471 (47.4%) | 232 (46.0%) | 0.34 |
|  | Female | 523 (52.6%) | 271 (53.8%) |  |
|  | Unknown | 0 (0.0%) | 1 (0.2%) |  |
| Race | African American | 134 (13.5%) | 75 (14.9%) | 0.20 |
|  | Asian | 30 (3.0%) | 9 (1.8%) |  |
|  | Caucasian | 703 (70.7%) | 342 (67.9%) |  |
| Ethnicity | Other/Unknown | 127 (12.8%) | 78 (15.5%) |  |
|  | Hispanic | 107 (10.8%) | 66 (13.1%) | 0.32 |
|  | Not Hispanic | 778 (78.3%) | 390 (77.4%) |  |
|  | Unknown | 109 (11.0%) | 48 (9.5%) |  |
| Region | Midwest | 347 (34.9%) | 169 (33.5%) | 0.83 |
|  | Northeast | 248 (24.9%) | 117 (23.2%) |  |
|  | Other/Unknown | 86 (8.7%) | 45 (8.9%) |  |
|  | South | 281 (28.3%) | 156 (31.0%) |  |
|  | West | 32 (3.2%) | 17 (3.4%) |  |
| BMI, mean (SD) |  | 30.9 (7.1) | 31.3 (6.9) | 0.29 |
| insurance | Commercial | 411 (41.3%) | 401 (79.6%) | 0.82 |
|  | Medicaid | 99 (10.0%) | 103 (20.4%) |  |
|  | Medicare | 335 (33.7%) | 345 (68.5%) |  |
|  | Other Payor Type | 32 (3.2%) | 159 (31.5%) |  |
|  | Uninsured | 36 (3.6%) | 383 (76.0%) |  |
|  | Unknown | 81 (8.1%) | 121 (24.0%) |  |
| Comorbidities | | | | |
| Myocardial infarction | Absent | 811 (81.6%) | 401 (79.6%) | 0.35 |
|  | Present | 183 (18.4%) | 103 (20.4%) |  |
| Congestive heart failure | Absent | 694 (69.8%) | 345 (68.5%) | 0.59 |
|  | Present | 300 (30.2%) | 159 (31.5%) |  |
| Peripheral vascular disease | Absent | 726 (73.0%) | 383 (76.0%) | 0.22 |
|  | Present | 268 (27.0%) | 121 (24.0%) |  |
| Cerebrovascular disease | Absent | 762 (76.7%) | 375 (74.4%) | 0.33 |
|  | Present | 232 (23.3%) | 129 (25.6%) |  |
| Dementia | Absent | 887 (89.2%) | 452 (89.7%) | 0.79 |
|  | Present | 107 (10.8%) | 52 (10.3%) |  |
| COPD | Absent | 619 (62.3%) | 302 (59.9%) | 0.38 |
|  | Present | 375 (37.7%) | 202 (40.1%) |  |
| Rheumatic diseases | Absent | 980 (98.6%) | 490 (97.2%) | 0.064 |
|  | Present | 14 (1.4%) | 14 (2.8%) |  |
| Peptic ulcer disease | Absent | 951 (95.7%) | 474 (94.0%) | 0.17 |
|  | Present | 43 (4.3%) | 30 (6.0%) |  |
| Mild liver disease | Absent | 854 (85.9%) | 436 (86.5%) | 0.75 |
|  | Present | 140 (14.1%) | 68 (13.5%) |  |
| Diabetes mellitus, uncomplicated | Absent | 637 (64.1%) | 308 (61.1%) | 0.26 |
|  | Present | 357 (35.9%) | 196 (38.9%) |  |
| Diabetes mellitus, complicated | Absent | 924 (93.0%) | 473 (93.8%) | 0.52 |
|  | Present | 70 (7.0%) | 31 (6.2%) |  |
| Kidney disease | Absent | 698 (70.2%) | 355 (70.4%) | 0.93 |
|  | Present | 296 (29.8%) | 149 (29.6%) |  |
| Malignancy | Absent | 825 (83.0%) | 421 (83.5%) | 0.79 |
|  | Present | 169 (17.0%) | 83 (16.5%) |  |
| Severe liver disease | Absent | 984 (99.0%) | 498 (98.8%) | 0.74 |
|  | Present | 10 (1.0%) | 6 (1.2%) |  |
| AIDS | Absent | 990 (99.6%) | 499 (99.0%) | 0.16 |
|  | Present | 4 (0.4%) | 5 (1.0%) |  |
| Metastatic solid tumor | Absent | 941 (94.7%) | 481 (95.4%) | 0.52 |
|  | Present | 53 (5.3%) | 23 (4.6%) |  |

Supplementary Table S3: Characteristics of all patients not hospitalized between day 0 to 7 post-infection included in propensity score analysis

|  |  | Not vaccinated  (n = 5544) | Vaccinated (n = 2802) | p-value |
| --- | --- | --- | --- | --- |
| Demographic variables | | | | |
| Patient age, mean (SD) |  | 49.1 (17.4) | 49.2 (17.0) | 0.70 |
| Gender | Male | 1968 (35.5%) | 1023 (36.5%) | 0.45 |
|  | Female | 3573 (64.4%) | 1776 (63.4%) |  |
|  | Unknown | 3 (0.1%) | 3 (0.1%) |  |
| Race | African American | 344 (6.2%) | 201 (7.2%) | 0.18 |
|  | Asian | 102 (1.8%) | 64 (2.3%) |  |
|  | Caucasian | 4012 (72.4%) | 1997 (71.3%) |  |
| Ethnicity | Other/Unknown | 1086 (19.6%) | 540 (19.3%) |  |
|  | Hispanic | 508 (9.2%) | 264 (9.4%) | 0.77 |
|  | Not Hispanic | 4259 (76.8%) | 2160 (77.1%) |  |
|  | Unknown | 777 (14.0%) | 378 (13.5%) |  |
| Region | Midwest | 2113 (38.1%) | 1028 (36.7%) | 0.59 |
|  | Northeast | 1460 (26.3%) | 751 (26.8%) |  |
|  | Other/Unknown | 698 (12.6%) | 342 (12.2%) |  |
|  | South | 977 (17.6%) | 524 (18.7%) |  |
|  | West | 296 (5.3%) | 157 (5.6%) |  |
| BMI, mean (SD) |  | 30.8 (6.1) | 30.8 (6.1) | 0.56 |
| insurance | Commercial | 3673 (66.3%) | 1796 (64.1%) | 0.38 |
|  | Medicaid | 282 (5.1%) | 148 (5.3%) |  |
|  | Medicare | 634 (11.4%) | 350 (12.5%) |  |
|  | Other Payor Type | 146 (2.6%) | 87 (3.1%) |  |
|  | Uninsured | 98 (1.8%) | 56 (2.0%) |  |
|  | Unknown | 711 (12.8%) | 365 (13.0%) |  |
| Comorbidities | | | | |
| Myocardial infarction | Absent | 5282 (95.3%) | 2660 (94.9%) | 0.49 |
|  | Present | 262 (4.7%) | 142 (5.1%) |  |
| Congestive heart failure | Absent | 5209 (94.0%) | 2629 (93.8%) | 0.81 |
|  | Present | 335 (6.0%) | 173 (6.2%) |  |
| Peripheral vascular disease | Absent | 5222 (94.2%) | 2638 (94.1%) | 0.93 |
|  | Present | 322 (5.8%) | 164 (5.9%) |  |
| Cerebrovascular disease | Absent | 5172 (93.3%) | 2607 (93.0%) | 0.67 |
|  | Present | 372 (6.7%) | 195 (7.0%) |  |
| Dementia | Absent | 5478 (98.8%) | 2764 (98.6%) | 0.52 |
|  | Present | 66 (1.2%) | 38 (1.4%) |  |
| COPD | Absent | 4313 (77.8%) | 2166 (77.3%) | 0.61 |
|  | Present | 1231 (22.2%) | 636 (22.7%) |  |
| Rheumatic diseases | Absent | 5492 (99.1%) | 2782 (99.3%) | 0.30 |
|  | Present | 52 (0.9%) | 20 (0.7%) |  |
| Peptic ulcer disease | Absent | 5416 (97.7%) | 2736 (97.6%) | 0.89 |
|  | Present | 128 (2.3%) | 66 (2.4%) |  |
| Mild liver disease | Absent | 5123 (92.4%) | 2594 (92.6%) | 0.78 |
|  | Present | 421 (7.6%) | 208 (7.4%) |  |
| Diabetes mellitus, uncomplicated | Absent | 4689 (84.6%) | 2355 (84.0%) | 0.53 |
|  | Present | 855 (15.4%) | 447 (16.0%) |  |
| Diabetes mellitus, complicated | Absent | 5438 (98.1%) | 2753 (98.3%) | 0.60 |
|  | Present | 106 (1.9%) | 49 (1.7%) |  |
| Kidney disease | Absent | 5190 (93.6%) | 2604 (92.9%) | 0.24 |
|  | Present | 354 (6.4%) | 198 (7.1%) |  |
| Malignancy | Absent | 5114 (92.2%) | 2561 (91.4%) | 0.18 |
|  | Present | 430 (7.8%) | 241 (8.6%) |  |
| Severe liver disease | Absent | 5527 (99.7%) | 2792 (99.6%) | 0.70 |
|  | Present | 17 (0.3%) | 10 (0.4%) |  |
| AIDS | Absent | 5528 (99.7%) | 2796 (99.8%) | 0.53 |
|  | Present | 16 (0.3%) | 6 (0.2%) |  |
| Metastatic solid tumor | Absent | 5452 (98.3%) | 2761 (98.5%) | 0.50 |
|  | Present | 92 (1.7%) | 41 (1.5%) |  |
